# Peritonsillar abscess in children: A retrospective analysis on surgical and antimicrobial approaches

**DOI:** 10.1371/journal.pone.0324276

**Published:** 2025-07-07

**Authors:** Stefan Alexander Rudhart, Thorsten Send, Maike Tilk, Barbara Leggewie, Franziska Bosse, Klaus Eichhorn, Sebastian Strieth, Stephan Hoch, Alexander Philippe Maas

**Affiliations:** 1 Department of Otorhinolaryngology, University Medical Center Bonn, Bonn, Germany; 2 Department of Otorhinolaryngology, Head and Neck Surgery, University Hospital Marburg, Philipps-Universität Marburg, Marburg, Germany; 3 Department for hearing disorders, tinnitus, vertigo and cochlear implants, MEDIAN Kaiserberg-Klinik, Bad Nauheim, Germany; University of Porto Faculty of Medicine, PORTUGAL

## Abstract

**Background:**

Peritonsillar abscess (PTA) is a prevalent infection for specialists in otorhinolaryngology and pediatric primary care providers, that has the potential to cause severe complications. The aim of this study is to investigate the surgical treatment of pediatric peritonsillar abscesses and to compare the risk profiles of bilateral surgery versus surgery on the affected side alone. In addition, the evaluation of the microbiological smears obtained intraoperatively should provide information on whether the calculated antibiotic therapy adequately covers the microbial spectrum.

**Methods:**

We conducted a retrospective analysis of pediatric patients (n = 150), who were treated for PTA between 2009 and 2024 by unilateral tonsillectomy (UTE) or bilateral tonsillectomy (BTE). Patient charts were analyzed regarding risk of bleeding, occurrence of other complications, recurrence rates in case of UTE as well as microbiological flora and antibiotic treatment.

**Results:**

Postoperative bleeding did not differ significantly between both groups. In 4.4% of the patients treated by UTE a recurrent PTA was found. No other severe complications after surgical treatment were found. Antibiotic treatment mainly relied on Cefuroxime and Ampicillin-Sulbactam, which is in accordance with the detected microbiological flora.

**Conclusions:**

No relevant differences were found with regard to the complication rate between UTE und BTE in pediatric patients. Broad-spectrum antibiotics were used in accordance with the detected microbiological flora. Since 2019, calculated antibiotic therapy with Ampicillin-Sulbactam has been the treatment of choice for pediatric PTA.

## Introduction

**Fig 1:**
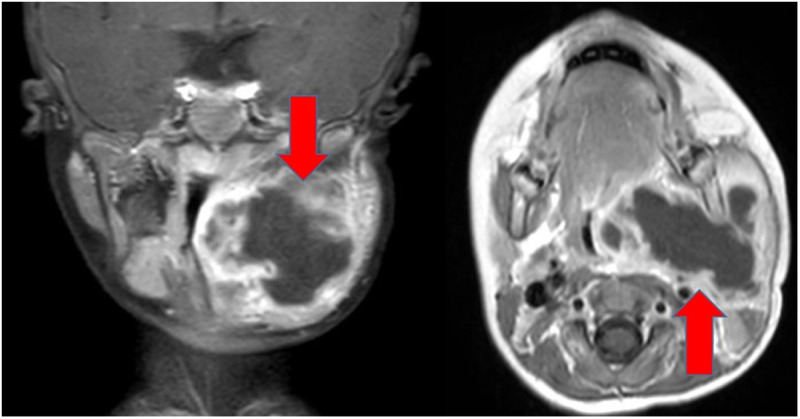
Parapharyngeal abscess in 19-month-old boy. Magnetic resonance imaging (MRI) with intravenous contrast medium of a 19-month-old boy with a parapharyngeal abscess (T1-weighted): The left image shows the coronal plane, the right image the axial plane. The red arrows mark the extensive parapharyngeal abscess on the left side (4.6 x 5.2 x 4.9 cm; cranial to caudal x right to left x ventral to dorsal) with inhomogeneous diffusion disturbance and marginal accumulation of contrast medium. The abscess extends around the mandible, is not directly related to the internal carotid artery and surrounds the external carotid artery. It extends cranially to the nasopharynx and reaches the thyroid gland caudally. The abscess formation displaces the oropharyngeal, hypopharyngeal and cervical structures to the opposite side, especially the trachea. The adjacent soft tissue structures show inflammatory edematous changes.

Peritonsillar abscess (PTA) is a prevalent deep infection with an incidence of 10–45 cases per 100.000 annually [1–3]. It represents a common clinical challenge not only for specialists in otorhinolaryngology but also for pediatric primary care providers, e.g., as a complication in case of scarlet fever [4]. The potential complications of PTA, including airway compromise, deep neck and chest soft tissue infections or mediastinitis underscore the significance of effective management strategies [1] (Fig 1). Traditionally, abscess tonsillectomy (TE) has been the preferred surgical intervention addressing PTA in children. However, the procedure is associated with notable risks, particularly secondary bleeding and the potential for bacteraemia, which could lead to systemic spread of infection and even endocarditis [5,6].

The effectiveness of alternative therapeutic approaches such as needle aspiration and incision with drainage under local anesthesia vary depending on the abscess’s location and other clinical factors [7,8]. Due to child incompliance these procedures cannot be considered in children [9]. Some studies suggest intravenous antibiotic therapy alone may suffice in select cases, although the recurrence rate and need for subsequent surgical intervention remain unclear [10,11]. To date, there have been divergent approaches regarding TE in the management of peritonsillar abscess. It remains unclear whether bilateral tonsillectomy (BTE), aimed at preventing contralateral recurrence of abscess, or a staged TE following recurrent acute tonsillitis adequately address the risk of complications associated with BTE [12,13]. In children, the situation is further complicated by potential hidden complications such as endocarditis, which is often detected later in pediatric cases [6,14]. Additionally, postoperative bleeding in children can have more severe consequences due to factors such as lack of compliance and lower blood volume reserves [6]. Thus far, according to the authors, there are no studies providing a basis for deciding between unilateral (UTE) or BTE for PTA in children. Recent literature indicates that there are differences in the pathogen spectrum in PTA depending on patient age, in particular that anaerobic bacteria are significantly more prevalent in patients over 50 years of age [15]. It should therefore be investigated further whether the spectrum of bacteria identified differs between children and adults, and how antibiotic treatment was conducted in children. As it is crucial to tailor treatment strategies based on age-specific microbial profiles to ensure optimal therapeutic outcomes in pediatric patients.

## Materials and methods

### Clinical data and inclusion criteria

This study conducts a retrospective analysis encompassing 150 pediatric patients (<18 years) treated at a quaternary university hospital spanning the last 15 years (2009–2024), with a follow up of at least 2 months.

The diagnosis of PTA was primarily made on the basis of clinical findings. In cases where the clinical findings were inconclusive, the diagnosis was supported by imaging procedures. In most cases, an ultrasound examination of the neck or, in selected cases, magnetic resonance imaging was performed. In order to avoid radiation exposure, computed tomography (CT) scans with intravenous contrast medium were generally not performed on underage patients. By evaluating the surgical reports, patients with a differential diagnosis of an intratonsillar abscess could be excluded.

Patients were categorized based on their treatment approach into a UTE group and a BTE group with abscess TE on the affected side and TE on the contralateral side or abscess TE on both sides in case of bilateral affection. Within the BTE group, patients with bilateral abscesses and patients with unilateral abscesses were compared.

The primary hypothesis posited that patients treated by UTE are more likely to develop a contralateral PTA or to undergo an elective TE because of recurrent acute tonsillitis of the remaining tonsil in the future. Additionally, the study compares the frequency of post-procedural bleeding among patients undergoing UTE or BTE.

Only patients under the age of 18 years were included in this study. Patients with intratonsillar abscess, peritonsillitis and patients who showed sufficient clinical improvement under antibiotic treatment without need for surgical intervention were excluded. Moreover, patients undergoing elective TE for recurrent acute tonsillitis and those with tonsil malignancies were also excluded from the analysis.

### Data collection

Patient records were retrospectively reviewed to collect general patients information (age, sex, date of hospitalization), specific treatment characteristics (histopathology, treatment, complications), and follow-up data (contralateral abscess, recurrent acute tonsillitis). This encompassed operation reports, notes of medical visits, physician letters, radiological imaging and laboratory findings. Patient consent for data analysis was obtained as per institutional guidelines.

The data were accessed for research purposes from March 21 to April 19, 2024. The data was blinded after extraction from the computer information system. The authors did not have access to information that could be used to identify individual participants after data collection. The study was conducted in the German state of North Rhine-Westphalia. According to the applying regional medical professional code of conduct, an ethics committee opinion does not have to be obtained for exclusively retrospective epidemiological research projects (see Professional Code of Conduct for Physicians in North Rhine, III § 15 (1)).

Regarding the necessity of a blood transfusion in the case of postoperative bleeding, the guidelines and recommendations of the German Society for Pediatric and Adolescent Medicine (DGKJ) were considered. These guidelines generally recommend a transfusion at a hemoglobin level below 7 g/dl and, depending on the relevant clinical and laboratory parameters, also between 8 and 10 g/dl. The decision regarding the necessity of a blood transfusion was made individually based on the affected patient.

### Follow-up

Daily medical ward rounds were carried out. All patients underwent at least four postoperative clinical examinations during their hospital stay. Additionally, they received at least one outpatient clinical follow-up visit, including inspection of the oral cavity and oropharynx.

As the study center is the only facility in the region that has the anesthesia resources necessary to treat pediatric PTA patients, it serves as the primary center for such cases via the emergency service. Therefore, we assume that a contralateral peritonsillar abscess would most likely have been treated at our facility again, unless the family had moved or intentionally sought treatment at another supra-regional center.

### Statistical analysis

Excel 2019 (Microsoft Corporation, Redmond, Washington, USA) was used for statistical descriptive analysis. Further statistical analyses were performed with IBM SPSS^®^ Statistics Version 28.0.0.0 (IBM Corp., Armonk, NY, USA). In general, for analysis of categorical data implemented in contingency tables a chi-squared test was performed. For smaller sample sizes Fisher´s exact test was employed. *P*-values < .05 were considered statistically significant.

## Results

During the study period from March 1, 2009 to March 1, 2024, 150 pediatric patients underwent either unilateral abscess TE (n = 92) or abscess TE with TE of the contralateral side (n = 53) or bilateral abscess TE (n = 5) at the University Hospital Bonn. 77 of these patients were male (51.3%) and 73 female (48.7%). The mean age of the cohort was 12.3 years, the youngest patient was three years and the oldest 17 years old. Concerning the side of PTA, the right side was affected 75 times (50.0%), the left side 70 times (46.7%) and in 5 cases (3.3%) both sides were affected. In 13 cases, the abscess cavity showed a clear peri- or retrotonsillar extension. The average duration of hospitalization was 4.4 days. It is worth mentioning that by September 2013, all of the 29 abscess TEs performed since March 1, 2009 had been performed bilaterally. We analyzed the distribution of pediatric PTA cases by month over the course of the study period. The highest number of cases was observed in May (n = 19) and October (n = 18), while the lowest incidence occurred in December (n = 8). Monthly case numbers ranged between 9 and 12 in February (n = 9), March (n = 12), April (n = 10), August (n = 10), September (n = 12), and November (n = 11). Slightly higher numbers were recorded in January (n = 13), June (n = 13), and July (n = 15). Although some variation between months is evident, we did not identify a clear seasonal trend or consistent peak in incidence.

### Complications

Postoperative bleeding occurred in 13.3% (20 of 150 patients) of all cases. Accordingly, 10.9% (10 of 92 patients) of unilaterally and 17.3% (10 of 58 patients) of bilaterally surgical treated patients were affected. This difference shows no statistical significance (chi-squared test; *p*-value: 0.264). Within the BTE group 1 of the 5 children with bilateral abscess was affected by postoperative bleeding, compared to 9 of 53 children with an unilateral abscess (Fisher’s exact test; p-value: 1). Comparing the bleeding risk of either of the two BTE subgroups with the UTE group there is also no significant difference.

Surgical intervention for postoperative bleeding was conducted in 9 of 10 bleeding incidents in the UTE group as well as in the BTE group. In neither the UTE nor the BTE group a transfusion of a red blood cell concentrate was necessary for any patient. Notable, one patient being diagnosed with a coagulation disorder did not suffer from postoperative bleeding.

Concerning patients of the BTE group with documented postoperative bleeding (n = 10) the contralateral side was affected by bleeding in 5 cases, while the ipsilateral side was affected in 2 cases. In one patient both sides were affected by bleeding and in 2 patients it cannot be withdrawn from the record, which side was affected. This difference in location of postoperative bleeding in the BTE group is statistically not significant (Fisher’s exact test; *p*-value: 0.453).

For patients of the UTE group in 4.4% of cases (4 out of 90) a contralateral abscess TE was conducted in the aftermath. These abscesses were diagnosed one month, nine months, 14 months and 38 months after the respective first PTA. In addition, in UTE group no further contralateral TE due to recurrent antibiotic-requiring tonsillitis was performed in the course of this study.

The occurrence of postoperative fever can only be reliably reported for 107 patients (71.3% of the study population; n = 85 UTE group; n = 22 BTE group). Two patients of the UTE group developed postoperative fever, while no patient of the BTE group was affected. This difference is statistically not significant (chi-squared test; *p*-value: 0.468).

Concerning the whole study population, there were no other major surgical or disease related complications identified in the record besides one patient of the UTE group, who developed persistent sore throat post intervention.

### Microbiological flora

The processing of the microbiological samples taken intraoperatively (n = 131) yielded the following result: 34 samples (26.0%) showed the presence of upper respiratory tract flora without further specification. In 44 cases (33.6%), upper respiratory tract flora and at least one specific bacterial strain was reported. Table 1 lists all bacterial strains detected by intraoperative sampling sorted according to frequency. By sampling the most frequently detected bacteria strain was Streptococcus pyogenes in 40 cases, bacteria of the Streptococcus mitis group in 15 cases, Staphylococcus aureus in 10 cases, Prevotella melaninogenica in 10 cases, Haemophilus influenzae in 9 cases, Fusobacterium necrophorum in 8 cases, Neisseria flava in 7 cases, Viridans streptococci in 6 cases and Streptococcus dysgalactiae in 5 cases. In 19 out of 150 cases, no result of the microbiological processing could be taken from the data.

**Table 1:** Results of intraoperatively taken microbiological samples.

Number of specific detections	Identified bacteria
40	Streptococcus pyogenes
15	Streptococcus mitis
10	Staphylococcus aureus
10	Prevotella melaninogenica
9	Haemophilus influenzae
8	Fusobacterium necrophorum
7	Neisseria flava
6	Not further sepcified viridans streptococci
5	Streptococcus dysgalactiae
4	Not further specified haemolytic streptococci
4	Haemophilus parainfluenzae
3	Streptococcus constellatus
3	Streptococcus parasanguinis
3	Neisseria mucosa
7	Other bacteria, each 2 times detected
8	Other bacteria, each 1 time detected

### Antibiotic treatment

**Fig 2:**
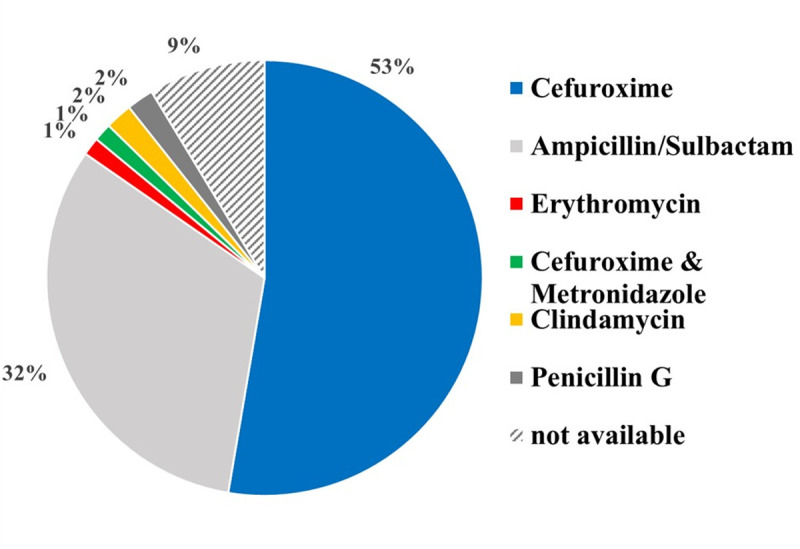
Peri- and postoperative antibiotic regimen over the whole study period.

In 13 of the 150 cases, the antibiotic therapy cannot be evaluated due to the retrospective setting of this study. Antibiotic therapies were age- and weight-adapted. With regard to the calculated antibiotic treatment regimen, Cefuroxime (79 out of 137) was used most frequently, followed by Ampicillin-Sulbactam (48 out of 137). In contrast, other intravenous antibiotic therapies were only prescribed in individual cases. Three patients each were treated with Clindamycin or Penicillin G and two patients each were treated with Erythromcyin or the combination of Cefuroxime and Metronidazole (Fig 2).

**Fig 3:**
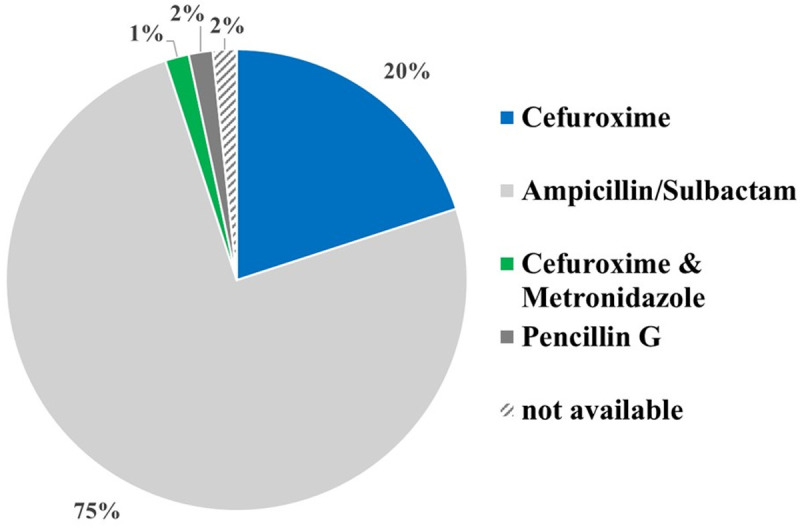
Peri- and postoperative antibiotic regimen after clinic intern change in antibiotic regimen towards Ampicillin-Sulbactam.

Although less frequently prescribed over the entire observation period, Ampicillin-Sulbactam became the regimen of choice for the calculated antibiotic treatment of pediatric peritonsillar abscess at the study center in late 2019. As of this period, 75% of patients (45 out of 60) in the study center received Ampicillin-Sulbactam as calculated antibiotic therapy (Fig 3).

## Discussion

PTA presents a significant clinical challenge due to its potential complications, including airway compromise and mediastinitis [1]. While abscess TE has traditionally been the preferred surgical intervention, it carries inherent risks such as secondary bleeding and bacteraemia [5].

There is an ongoing and clinically relevant debate regarding the appropriate surgical management of pediatric PTA. While abscess drainage under local anesthesia is widely accepted as the treatment of choice in adult patients, its applicability in pediatric cases remains limited, primarily due to issues of patient compliance and tolerability [9].

A retrospective single-center study by Graham et al., which included 188 pediatric cases, provides valuable insights into this issue [16]. The authors demonstrated that abscess drainage can be safely performed in children when appropriate anesthetic strategies are applied. The study compared outcomes among awake patients, those under conscious sedation, and those receiving general anesthesia. However, it is worth noting that long-term outcomes—particularly regarding recurrence rates and the need for re-hospitalization—were not comprehensively addressed.

Further supporting this discussion, the recent multicenter study by Rosi-Schumacher et al., involving 777 pediatric patients, compared abscess drainage with abscess TE [17]. The study found no significant differences in key outcome measures such as length of hospital stay, readmission rates, or the need for repeat surgical intervention. Specifically, 1.4% of patients initially treated with drainage required reoperation for drainage, and 0.83% underwent TE within a 30-day follow-up period.

In the case of very small abscesses, conservative treatment with intravenous antibiotics may also be considered in children. However, the data on this approach is insufficient, and recurrence of the abscess or of other complications appears to be higher [10,11].

With 150 cases, this study is one of the largest single-center studies on pediatric PTA. Our study aims to address the optimal management strategy for PTA in children, focusing on the choice between UTE and BTE. We observed that UTE was the predominant approach, with a notable proportion of 4.4% of patients exhibiting contralateral abscess development requiring subsequent abscess TE. Due to the retrospective nature of our study, this information, if the patients were affected by recurrent tonsillitis, it is not comprehensively available for all cases due to gaps in documentation. However, it is noteworthy that, until September 2013, BTE was the standard surgical approach in our institution, regardless of the history of recurrent tonsillitis.

Postoperative bleeding was a common complication, occurring in both unilateral and bilateral TE groups, although the difference was not statistically significant. In literature the haemorrhage risk after TE is mentioned with 2.72% to 6.93% in cases of TE without acute infection [18]. However, the haemorrhage risk in case of PTA in pediatric patients is unknown yet. In adult patients the bleeding risk is around 3.6% to 12% [12,13,19–23]. TE appears to be less painful for children than for adults [24].

Postoperative bleeding in the BTE group often occurred on the contralateral side, suggesting potential implications for surgical technique. These findings are consistent with the German guideline for TE, which tends to lean towards a more conservative indication for contralateral TE [25,26]. However, further investigation is warranted to elucidate these findings fully. Our data also supports this shift, showing management moving away from BTE in unilateral PTA starting from the year 2013. Currently, our institutional standard is to offer parents of children with a documented history of recurrent tonsillitis (defined as ≥6 episodes per year requiring antibiotic treatment) affected by PTA the option of BTE.

In this cohort no blood transfusion was reported, taking into account the guidelines and recommendations of the German Society for Pediatric and Adolescent Medicine (DGKJ) [27].

Regarding the occurrence of postoperative fever, our findings did not reveal a significant difference between the UTE and BTE group. While no major complications were identified in our study cohort, one patient undergoing UTE reported persistent sore throat post-intervention, highlighting the importance of thorough postoperative evaluation and patient follow-up. Postoperative fever after TE in children can be influenced by various risk factors. Infections by bacterial or viral pathogens during or after the surgery are significant risk factors [28]. Additionally, the surgical procedure itself can trigger a local inflammatory reaction in the operated area, leading to systemic effects resulting in fever [29]. Children with a weakened immune system or pre-existing conditions are particularly susceptible to postoperative complications, including fever [30]. The duration and complexity of the surgery also play a role, as longer and more complex procedures increase the risk of complications and infections, which in turn can cause fever [31]. Further, inadequate postoperative care and hygiene can also promote infections. Proper wound care and continuous monitoring are therefore essential to prevent infections. Another risk factor is dehydration, as difficulties in food and fluid intake after surgery can impair the healing process and lead to secondary infections and fever [32].

Overall, the detected bacteria strains are consistent with those commonly reported in PTA. Streptococcus species (especially Streptococcus pyogenes), anaerobes like Fusobacterium necrophorum, and Staphylococcus aureus are well-documented pathogens in these infections [33]. Some of the bacteria identified in the study are more likely to be part of the physiological mucosal flora. These include the Streptococcus mitis group, Neisseria flava and Viridans streptococci. However, they can sometimes act as opportunistic pathogens if they invade sterile areas or if the host’s immune defenses are weakened.

Among the bacteria identified in the study, Haemophilus influenzae was detected in 9 cases. This finding prompts a discussion on the potential role of vaccination in preventing infections caused by Haemophilus influenzae. In this pediatric study group, it is pertinent to consider many of these children may not have been vaccinated against Haemophilus influenzae, especially if they were younger and had not completed their routine childhood immunizations. Due to the retrospective nature of our study, individual vaccination records were not consistently documented in the patients’ medical histories. As a result, we are unable to determine with certainty whether the children affected by Haemophilus influenzae were vaccinated. The Haemophilus influenzae type b (Hib) vaccine is typically administered during infancy as part of routine childhood vaccinations [34]. This vaccine has been highly effective in reducing the incidence of invasive Hib caused diseases, including meningitis and bacteremia in vaccinated populations. However, coverage rates can vary depending on healthcare access, vaccination policies, and individual compliance with immunization schedules [34]. Given the presence of Haemophilus influenzae in cases of PTA identified in the study, there is a potential role for vaccination in preventing PTA in children.

With regard to Streptococcus pyogenes (Group A Streptococcus, Strep A), which has 40 times been detected in our study cohort of 150 patients, no licensed vaccine currently exists, despite decades of research and the significant global disease burden associated with this pathogen [35–37]. Our study highlights that the successful introduction of a future Strep A vaccine could potentially significantly reduce the incidence of PTA in children by reducing primary streptococcal infections.

For Streptococcus pneumoniae routine vaccination in Germany includes immunization. The Standing Committee on Vaccination (STIKO) at the Robert Koch Institute recommends the pneumococcal conjugate vaccine (PCV13) for all infants as part of the standard immunization schedule. For specific risk groups—such as individuals with immunosuppression or chronic illnesses—a sequential vaccination with PCV13 followed by the polysaccharide vaccine PPSV23 is advised [38–40].

The analysis of the antibiotic treatment regimens for pediatric PTA highlights significant findings regarding the preferred antibiotics and their alignment with the identified bacterial pathogens. Cefuroxime was the most frequently used antibiotic, administered in 79 out of 137 cases, followed by Ampicillin-Sulbactam, prescribed in 48 out of 137 cases. This pattern reflects a strategic approach to target the most common bacterial pathogens identified in PTA. The most frequently detected bacteria included Streptococcus pyogenes, Streptococcus mitis group, Staphylococcus aureus, Prevotella melaninogenica, Haemophilus influenzae, and Fusobacterium necrophorum. Because of its broad-spectrum activity against many of these pathogens, particularly Streptococcus pyogenes and Staphylococcus aureus Cefuroxime is the most frequently used empirical antibiotic in this study. Cefuroxime’s efficacy against both gram-positive and gram-negative bacteria makes it a suitable empirical choice in treating these infections [41,42]. Ampicillin-Sulbactam emerged as the preferred antibiotic in late 2019, with 75% of patients (45 out of 60) receiving it. The shift in our antibiotic protocol was based on the 2019 S2k guideline on antibiotic therapy for ENT infections, published by the German Society of Oto-Rhino-Laryngology, Head and Neck Surgery (DGHNO-KHC). This guideline recommends Ampicillin-Sulbactam as the empiric first-line treatment for peritonsillar abscesses. In response to this updated national recommendation, our clinic revised its internal treatment standard accordingly.

Ampicillin-Sulbactam is effective against a range of bacteria, including Streptococcus pyogenes, Streptococcus mitis, and anaerobes like Prevotella melaninogenica and Fusobacterium necrophorum [43]. The addition of Sulbactam, a beta-lactamase inhibitor, enhances Ampicillin’s efficacy against beta-lactamase producing bacteria, providing a broader spectrum of activity and reducing the likelihood of resistance [44]. In pharmacotherapy, there has been a proven trend towards increased use of penicillin-based antibiotics to prevent bacterial resistance [45]. Penicillin G, Erythromycin, and the combination of Cefuroxime and Metronidazole were reserved for specific cases where bacterial profiles or patient conditions warranted their use. Clindamycin was primarily used for patients with a Penicillin allergy, as Cefuroxime would not be suitable due to cross-allergies; described in 5–10% [46,47]. A careful medical history regarding Penicillin allergy is important, as many patients mistakenly believe they have a Penicillin allergy. Symptoms these patients attribute to an allergy are often side effects of previous Penicillin treatment rather than true allergic reactions [48]. However, current literature discusses whether additional antibiotic therapy is necessary following abscess TE for PTA, but this consideration has not yet been widely adopted [49]. Therefore, all patients evaluated in this study received intravenous antibiotics postoperatively. Future studies should continue to monitor antibiotic efficacy and resistance trends to further refine treatment protocols.

Our study contributes valuable insights into the management of PTA in children, regarding the choice between UTE and BTE and calculated antibiotic treatment. However, studies with larger sample sizes and longer follow-up periods are needed to validate our findings and refine PTA treatment guidelines. Additionally, prospective studies examining the efficacy of alternative treatment modalities such as needle aspiration and incision drainage in pediatric patients could further inform clinical practice and optimize patient outcomes.

## Limitations

Despite presenting an extensive pediatric original cohort on PTA, this study is limited by its retrospective approach conducted at a single center. Documentation was occasionally incomplete and inconsistent, relying on surgical reports and records from colleagues. However, the study’s single-center design ensured uniform documentation practices and maintained consistency in therapeutic approaches.

## Conclusion

Although abscess TE an established surgical procedure for PTA, it carries risks such as secondary bleeding, meaning that abscess drainage must also be discussed on an individual basis in pediatric patients. Our study, focusing on UTE versus bilateral BTE in children, found that UTE was predominant, with notable cases of contralateral abscess or recurrent tonsillitis requiring subsequent surgery. Postoperative bleeding did not differ significantly between both groups. Antibiotic treatment mainly relied on Cefuroxime and Ampicillin-Sulbactam, reflecting their coverage against common pathogens like Streptococcus species and Staphylococcus aureus. Ampicillin-Sulbactam became favored from late 2019 onward due to its effectiveness and broader antimicrobial spectrum. Further research is needed to explore the necessity of postoperative antibiotic therapy and refine treatment protocols. Despite limitations like retrospective design and single-center data, our findings contribute valuable insights into optimizing PTA management in children. Prospective studies are needed to validate these findings and guide clinical practice effectively.

## Supporting information

S1 Table Tabular presentation of the data. (XLSX)

## References

[1] Galioto NJ. Peritonsillar abscess. Am Fam Physician. 2017;95(8):501–6. 28409615

[2] Sowerby LJ, Hussain Z, Husein M. The epidemiology, antibiotic resistance and post-discharge course of peritonsillar abscesses in London, Ontario. J Otolaryngol Head Neck Surg. 2013;42(1):5. doi: 10.1186/1916-0216-42-5 23663820 PMC3646551

[3] Wikstén J, Blomgren K, Eriksson T, Guldfred L, Bratt M, Pitkäranta A. Variations in treatment of peritonsillar abscess in four Nordic countries. Acta Otolaryngol. 2014;134(8):813–7. doi: 10.3109/00016489.2014.905702 24930914

[4] Powers GF, Boisvert PL. Age as a factor in streptococcosis. J Pediatr. 1944;25(6):481–504. doi: 10.1016/s0022-3476(44)80171-8

[5] Bannister M, Thompson C. Post-tonsillectomy dietary advice and haemorrhage risk: Systematic review. Int J Pediatr Otorhinolaryngol. 2017;103:29–31. doi: 10.1016/j.ijporl.2017.09.031 29224760

[6] Klug TE, Henriksen J-J, Rusan M, Fuursted K, Ovesen T. Bacteremia during quinsy and elective tonsillectomy: an evaluation of antibiotic prophylaxis recommendations for patients undergoing tonsillectomy. J Cardiovasc Pharmacol Ther. 2012;17(3):298–302. doi: 10.1177/1074248411423023 22026972

[7] Chang BA, Thamboo A, Burton MJ, Diamond C, Nunez DA. Needle aspiration versus incision and drainage for the treatment of peritonsillar abscess. Cochrane Database Syst Rev. 2016;12(12):CD006287. doi: 10.1002/14651858.CD006287.pub4 28009937 PMC6463807

[8] Ali SA, Kovatch KJ, Smith J, Bellile EL, Hanks JE, Truesdale CM, et al. Predictors of intratonsillar versus peritonsillar abscess: A case-control series. Laryngoscope. 2019;129(6):1354–9. doi: 10.1002/lary.27615 30569506 PMC6755033

[9] Bauer PW, Lieu JE, Suskind DL, Lusk RP. The safety of conscious sedation in peritonsillar abscess drainage. Arch Otolaryngol Head Neck Surg. 2001;127(12):1477–80. doi: 10.1001/archotol.127.12.1477 11735818

[10] Battaglia A, Burchette R, Hussman J, Silver MA, Martin P, Bernstein P. Comparison of medical therapy alone to medical therapy with surgical treatment of peritonsillar abscess. Otolaryngol Head Neck Surg. 2018;158(2):280–6. doi: 10.1177/0194599817739277 29110574

[11] Souza DLS, Cabrera D, Gilani WI, Campbell RL, Carlson ML, Lohse CM, et al. Comparison of medical versus surgical management of peritonsillar abscess: A retrospective observational study. Laryngoscope. 2016;126(7):1529–34. doi: 10.1002/lary.25960 27010228

[12] Paradise JL, Bluestone CD, Bachman RZ, Colborn DK, Bernard BS, Taylor FH, et al. Efficacy of tonsillectomy for recurrent throat infection in severely affected children. Results of parallel randomized and nonrandomized clinical trials. N Engl J Med. 1984;310(11):674–83. doi: 10.1056/NEJM198403153101102 6700642

[13] Paradise JL, Bluestone CD, Colborn DK, Bernard BS, Rockette HE, Kurs-Lasky M. Tonsillectomy and adenotonsillectomy for recurrent throat infection in moderately affected children. Pediatrics. 2002;110(1 Pt 1):7–15. doi: 10.1542/peds.110.1.7 12093941

[14] Chandnani HK, Jain R, Patamasucon P. Group C streptococcus causing rheumatic heart disease in a child. J Emerg Med. 2015;49(1):12–4. doi: 10.1016/j.jemermed.2014.12.057 25797932

[15] Slouka D, Hanakova J, Kostlivy T, Skopek P, Kubec V, Babuska V, et al. Epidemiological and microbiological aspects of the peritonsillar abscess. Int J Environ Res Public Health. 2020;17(11):4020. doi: 10.3390/ijerph17114020 32516939 PMC7312574

[16] Graham ME, Neal AK, Newberry IC, Firpo MA, Park AH. Conscious sedation for pediatric peritonsillar abscess: Comparison of anesthetic approaches. Otolaryngol Head Neck Surg. 2019;160(4):706–11. doi: 10.1177/0194599818821905 30598050

[17] Rosi-Schumacher M, Nagy R, Virgen C, Carr MM. Peritonsillar abscess on NSQIP: Safety of indicated quinsy tonsillectomy. Int J Pediatr Otorhinolaryngol. 2023;171:111636. doi: 10.1016/j.ijporl.2023.111636 37352593

[18] Gutierrez JA, Shannon CM, Nguyen SA, Labadie RF, White DR. The impact of surgical indication on posttonsillectomy hemorrhage: A systematic review and meta-analysis. Otolaryngol Head Neck Surg. 2023;169(4):780–91. doi: 10.1002/ohn.339 37003296

[19] Ketterer MC, Maier M, Burkhardt V, Mansour N, Knopf A, Becker C. The peritonsillar abscess and its management - is incision and drainage only a makeshift to the tonsillectomy or a permanent solution?. Front Med (Lausanne). 2023;10:1282040. doi: 10.3389/fmed.2023.1282040 38093972 PMC10716296

[20] Koskenkorva T, Koivunen P, Penna T, Teppo H, Alho O-P. Factors affecting quality-of-life impact of adult tonsillectomy. J Laryngol Otol. 2009;123(9):1010–4. doi: 10.1017/S0022215109005271 19389265

[21] Alho O-P, Koivunen P, Penna T, Teppo H, Koskela M, Luotonen J. Tonsillectomy versus watchful waiting in recurrent streptococcal pharyngitis in adults: randomised controlled trial. BMJ. 2007;334(7600):939. doi: 10.1136/bmj.39140.632604.55 17347187 PMC1865439

[22] van Staaij BK, van den Akker EH, Rovers MM, Hordijk GJ, Hoes AW, Schilder AGM. Effectiveness of adenotonsillectomy in children with mild symptoms of throat infections or adenotonsillar hypertrophy: open, randomised controlled trial. BMJ. 2004;329(7467):651. doi: 10.1136/bmj.38210.827917.7C 15361407 PMC517640

[23] Hahn J, Barth I, Wigand MC, Mayer B, Hoffmann TK, Greve J. The surgical treatment of peritonsillar abscess: A retrospective analysis in 584 patients. Laryngoscope. 2021;131(12):2706–12. doi: 10.1002/lary.29677 34111309

[24] Esler M. The sympathetic system and hypertension. Am J Hypertens. 2000;13(6 Pt 2):99S-105S. doi: 10.1016/s0895-7061(00)00225-9 10921528

[25] Windfuhr JP, Schmuker C, Günster C. Sore throat as indication for tonsillectomy before and after implementation of the German guideline for tonsillitis : Longitudinal study covering 115.839 procedures. HNO. 2021;69(9):742–9. doi: 10.1007/s00106-020-00944-8 32945897

[26] Entzündliche Erkrankungen der Gaumenmandeln/ Tonsillitis, Therapie, (2019).

[27] Kulozik AE, Kunz J. Anämiediagnostik im Kindesalter. S1-Leitline der Gesellschaft für Pädiatrische Onkologie und Hämatologie – Version 4/2012. 2013;13(02):120–6. doi: 10.1055/s-0038-1629329

[28] Walsh RM, Kumar BN, Tse A, Jones PW, Wilson PS. Post-tonsillectomy bacteraemia in children. J Laryngol Otol. 1997;111(10):950–2. doi: 10.1017/s0022215100139040 9425484

[29] Corkum KS, Hunter CJ, Grabowski JE, Lautz TB. Early postoperative fever workup in children: utilization and utility. J Pediatr Surg. 2018;53(7):1295–300. doi: 10.1016/j.jpedsurg.2017.06.019 28693850

[30] Perlino CA. Postoperative fever. Med Clin North Am. 2001;85(5):1141–9. doi: 10.1016/s0025-7125(05)70369-2 11565491

[31] Lenhardt R, Negishi C, Sessler DI. Perioperative fever. Acta Anaesthesiol Scand Suppl. 1997;111:325–8. 9421064

[32] Canavan A, Arant BS Jr. Diagnosis and management of dehydration in children. Am Fam Physician. 2009;80(7):692–6. 19817339

[33] Raju G, Selvam EM. Evaluation of microbial flora in chronic tonsillitis and the role of tonsillectomy. Bangladesh J Otorhinolaryngol. 2012;18(2):109–13. doi: 10.3329/bjo.v18i2.11982

[34] Impfkommission S. Empfehlungen der Ständigen Impfkommission beim Robert Koch-Institut – 2019/2020. Epidemiol Bull. 2019;(34):313–64. doi: 10.25646/6233.7

[35] Steer AC, Batzloff MR, Mulholland K, Carapetis JR. Group A streptococcal vaccines: facts versus fantasy. Curr Opin Infect Dis. 2009;22(6):544–52. doi: 10.1097/QCO.0b013e328332bbfe 19797947

[36] Castro SA, Dorfmueller HC. A brief review on Group A Streptococcus pathogenesis and vaccine development. R Soc Open Sci. 2021;8(3):201991. doi: 10.1098/rsos.201991 33959354 PMC8074923

[37] Fan J, Toth I, Stephenson RJ. Recent scientific advancements towards a vaccine against Group A streptococcus. Vaccines (Basel). 2024;12(3):272. doi: 10.3390/vaccines12030272 38543906 PMC10974072

[38] Schley K, Borchert K, Seidel K, Jacob C, von Eiff C, Laurenz M. Did the change of the vaccination schedule effect pneumococcal conjugate vaccination compliance and adherence of premature and mature born infants in Germany? Answers from a claims database analysis. Vaccine. 2023;41(28):4081–91. doi: 10.1016/j.vaccine.2023.05.045 37263871

[39] Deb A, Podmore B, Barnett R, Beier D, Galetzka W, Qizilbash N, et al. Pneumococcal vaccination coverage in individuals (16-59 years) with a newly diagnosed risk condition in Germany. BMC Infect Dis. 2022;22(1):753. doi: 10.1186/s12879-022-07736-1 36171549 PMC9517976

[40] Rademacher J. Current and new vaccines against pneumococci. Inn Med (Heidelb). 2024;65(11):1076–81. doi: 10.1007/s00108-024-01766-4 39222146

[41] Perry CM, Brogden RN. Cefuroxime axetil. A review of its antibacterial activity, pharmacokinetic properties and therapeutic efficacy. Drugs. 1996;52(1):125–58. doi: 10.2165/00003495-199652010-00009 8799689

[42] Neu HC, Fu KP. Cefuroxime, a beta-lactamase-resistant cephalosporin with a broad spectrum of gram-positive and -negative activity. Antimicrob Agents Chemother. 1978;13(4):657–64. doi: 10.1128/AAC.13.4.657 248268 PMC352306

[43] Finegold SM. In vitro efficacy of β-lactam/ β-lactamase inhibitor combinations against bacteria involved in mixed infections. Int J Antimicrob Agents. 1999;12(Suppl 1):S9–14; discussion S26-7. doi: 10.1016/s0924-8579(99)00086-2 10526868

[44] Lode HM. Rational antibiotic therapy and the position of ampicillin/sulbactam. Int J Antimicrob Agents. 2008;32(1):10–28. doi: 10.1016/j.ijantimicag.2008.02.004 18539004

[45] Chatzopoulou M, Reynolds L. Role of antimicrobial restrictions in bacterial resistance control: a systematic literature review. J Hosp Infect. 2020;104(2):125–36. doi: 10.1016/j.jhin.2019.09.011 31542456

[46] Pichichero ME. Use of selected cephalosporins in penicillin-allergic patients: a paradigm shift. Diagn Microbiol Infect Dis. 2007;57(3 Suppl):13S-18S. doi: 10.1016/j.diagmicrobio.2006.12.004 17349459

[47] D’Errico S, Frati P, Zanon M, Valentinuz E, Manetti F, Scopetti M, et al. Cephalosporins’ cross-reactivity and the high degree of required knowledge. Case report and review of the literature. Antibiotics (Basel). 2020;9(5):209. doi: 10.3390/antibiotics9050209 32344946 PMC7277108

[48] Caubet J-C, Kaiser L, Lemaître B, Fellay B, Gervaix A, Eigenmann PA. The role of penicillin in benign skin rashes in childhood: A prospective study based on drug rechallenge. J Allergy Clin Immunol. 2011;127(1):218–22. doi: 10.1016/j.jaci.2010.08.025 21035175 PMC7126001

[49] Dhiwakar M, Eng CY, Selvaraj S, McKerrow WS. Antibiotics to improve recovery following tonsillectomy: A systematic review. Otolaryngol Head Neck Surg. 2006;134(3):357–64. doi: 10.1016/j.otohns.2005.12.016 16500427

